# Treatment with echinocandins during continuous renal replacement therapy

**DOI:** 10.1186/cc13803

**Published:** 2014-03-28

**Authors:** Francisco Javier González de Molina, Maria de Los Ángeles Martínez-Alberici, Ricard Ferrer

**Affiliations:** 1 Intensive Care Department, Hospital Universitari Mútua de Terrassa, Universitat de Barcelona, Plaça Dr. Robert, E-08221 Terrassa, Spain

## Abstract

Echinocandins are indicated as first-line treatment for invasive candidiasis in moderate to severe illness. As sepsis is the main cause of acute kidney injury, the combination of echinocandin treatment and continuous renal replacement therapy (CRRT) is common. Optimizing antibiotic dosage in critically ill patients receiving CRRT is challenging. The pharmacokinetics of echinocandins have been studied under various clinical conditions; however, data for CRRT patients are scarce. Classically, drugs like echinocandins with high protein binding and predominantly non-renal elimination are not removed by CRRT, indicating that no dosage adjustment is required. However, recent studies report different proportions of echinocandins lost by filter adsorption. Nevertheless, the clinical significance of these findings remains unclear.

## Review

## Introduction

Echinocandins are the first systemic antifungal agents that selectively target the fungal cell [1]. Three echinocandins are currently available: caspofungin, anidulafungin, and micafungin [2]. Echinocandins are cyclic hexapeptides with N-linked acyl lipid side chains. These lipophilic side chains predominantly differentiate the three echinocandins, resulting in variations in solubility, microbiological potency, and pharmacokinetic disposition [3-5]. Echinocandins are indicated as first-line antifungal treatment for invasive candidiasis in patients with moderately severe to severe illness [6].

The mechanism of action of the echinocandins is the non-competitive inhibition of β-(1,3)-D-glucan synthase [7], an essential component of many fungal cell walls, leading to osmotic instability and cell death [5,8]. Echinocandins exhibit fungistatic activity against *Aspergillus* species and concentration-dependent fungicidal activity against *Candida* species with a prolonged post-antifungal effect [9-11]. Acquired resistance to the echinocandins remains sporadic [12]. Both maximum concentration/minimum inhibitory concentration (C_max_/MIC) and AUC_0-24_/MIC (area under the curve over 24-hour dosing interval/MIC) have been associated with efficacy [13-15]. All echinocandins are highly protein-bound and have a low to moderate volume of distribution and very low excretion as unchanged drug in the urine [14,16]. Echinocandins have an excellent safety profile and low potential for pharmacological interactions [17,18].

Acute renal failure is a common complication of critical illness and carries a high mortality [19,20]. It affects up to 20% of critically ill patients with severe sepsis or septic shock [21-23]. These patients usually receive continuous renal replacement therapy (CRRT) and antibiotics simultaneously [24,25]. Antimicrobial pharmacokinetics are affected by CRRT and may be altered by acute renal failure and critical illness [26]. Several factors should be considered in antibiotic prescription under CRRT: pharmacokinetics, causative microorganism, MIC, renal replacement therapy mode, membrane and surface area, sieving coefficient, effluent and dialysate rates, and others. Only the unbound fraction of a drug is available for filtration, and drugs with high protein binding are largely unaffected by CRRT. The degree of protein binding is the most important factor influencing whether a drug needs dose adjustment during CRRT. Drugs that are more than 90% bound to plasma proteins are unlikely to be removed by hemodialysis and hemofiltration [27-31], but the adsorption on the membrane in the filter could result in a significant clearance [32-35].

## Drug adsorption clearance in continuous renal replacement therapy

Adsorption of antibiotics onto CRRT filters may result in drug elimination [36-44]. Most research on adsorption to CRRT filters has focused on the cytokines [45-52]. Few studies have investigated the adsorption of antibiotics onto CRRT filters, so the clinical importance of this phenomenon is unknown and is not taken into account in drug guidelines [27,30,31,53,54] or in clinical pharmacokinetic/pharmacodynamic (pK/pD) studies [55].

Adsorptive clearances depend on the selective or non-selective binding of molecules to the filter membrane. The adsorptive capacity for low-molecular-weight plasma proteins varies among different hemofilter membrane materials [35,56,57]. Adsorptive clearances for middle molecules are time-dependent, with maximum values occurring in the first few hours of therapy [58]. Adsorption onto filters is a saturable process that varies according to the timing of the filter changes, so that adsorption immediately after a filter change will be greater than 48 hours after a filter change. Thus, measuring drug concentrations close to the time of a filter change may alter estimates of clearance and should be avoided. If adsorption is reversible when circulating drug concentrations fall, the maximum adsorption during CRRT will be underestimated.

## Effect of membrane characteristics

The membranes used in CRRT are high-flux synthetic membranes, mainly made of polysulfone, polyamide, polyacrylonitrile (PAN), or polymethyl methacrylate. These membranes can be asymmetric or symmetric, and their thickness ranges from 40 to 100 μm. Hemofilter membranes made of PAN or polymethyl methacrylate are symmetric in structure, whereas those made from polysulfone or polyamide are asymmetric [59,60]. All these synthetic membranes are hydrophobic, and their pores allow passage of molecules ranging from 10 to 30,000 daltons. This pore size allows a high rate of ultrafiltration under relatively small pressures. These membranes have high sieving coefficient/saturation coefficients (S_c_/S_d_) for solutes in a wide range of molecular weights (0 to 20,000 daltons), and this further contributes to high drug and metabolite clearance [59,61]. Another aspect that influences adsorption is the membrane’s electrical charge [57]. Whereas polysulfone and polyamine hemofilters have no net charge, PAN filters have a negative charge. This last characteristic of hemofilters may influence adsorption and retention of drugs to negatively charged hemodialyzer membranes [62,63]. Moreover, if adsorption is due to ion interaction between the antibiotic and hemofilter, pH can affect drug adsorption to some filter membranes; for example, aminoglycosides and levofloxacin are significantly adsorbed by PAN membranes [38-40]. The extent of drug adsorption also depends on hemofilter surface area. It is reasonable to think that increasing surface area will increase the amount of drug adsorbed and may also increase the time to maximum adsorption. Adsorption to hemofilters varies with the materials used in their membranes. Different hollow fibers have different adsorptive capacities. Some filter membranes, such as those made from the commonly used PAN, may adsorb a substantial amount of drug to their surface compared with polyamine filter membranes [37,64].

## Effect of albumin on drug adsorption

Antibiotics vary largely in protein binding [65,66]. While aminoglycosides bind poorly to proteins, the percentage of echinocandins bound to proteins in serum exceeds 90%. The binding capacity of albumin is decreased not only in chronic kidney disease but also in acute renal failure. Other unknown factors include the effect of increased volume of distribution because of edema in critically ill patients and the possible impact of albumin concentration and protein binding on adsorption.

## Effect of continuous renal replacement therapy mode and flows

Transmembrane pressure may be an important determinant of adsorption. Membrane pores are structured like ‘water channels’. When the transmembrane pressure increases, the channels may open, resulting in an increase in the number of potential adsorptive sites [67,68]. Finally, the effects on adsorption of possible interactions between drugs and acute-phase proteins such as α1-acid glycoprotein, cytokines, and complement factors are unknown.

## Studies on echinocandins and continuous renal replacement therapy

### Caspofungin

Weiler and colleagues [42] have recently investigated the influence of continuous venovenous hemofiltration (CVVHF) and continuous venovenous hemodialysis (CVVHD) on the pharmacokinetics of caspofungin in critically ill patients in order to assess the appropriateness of standard dosage during CRRT (Table 1). The effects of CVVHD and CVVHF on caspofungin clearance, in order to detect an eventual adsorption, were assessed by determination of S_c_, S_d_, and the difference between caspofungin concentrations in the hemofilter/dialyzer inlet and outlet (C_in_ − C_out_). CRRT was performed with polysulfone hemofilters (0.71 m^2^ for CVVHF and 1.8 m^2^ for CVVHD). Sampling was performed on day 1 of caspofungin treatment (single dose) and at steady state on day 4 or later. Blood samples were drawn from an arterial line at 1, 2, 4, 8, 12, and 24 hours after the start of caspofungin infusion. For patients on CRRT, ultrafiltrate/dialysate samples were taken simultaneously. In addition, blood samples from the C_in_ − C_out_ were collected at 1 and 24 hours. Caspofungin was quantified in plasma samples by a liquid chromatography/mass spectroscopy method (online SPE-LC-MS/MS). Thirty-six plasma sample sets were obtained from 27 patients (14 patients on CRRT and 13 patients off CRRT). Assessment under both single-dose and steady-state conditions was possible for only eight patients. Six sets of ultrafiltrate samples were drawn from patients on CVVHF. Ultrafiltrate concentrations amounted to only 0.33% of the respective plasma levels; that is, the median (range) S_c_ was 0.0033 (0.0157). The hemofiltration clearance (CLHF) calculated from S_c_ was as low as 8.0 (37.8) mL/hour (1.8% (9.6%) of total clearance). When estimated from C_in_ − C_out_, the CLHF was higher (61.0 (10,712.2) mL/hour). Dialysate sampling was performed during all 11 sampling periods in patients on CVVHD. The S_d_ was 0.0027 (0.2470), yielding a hemodialysis clearance (CLHD) of 5.9 (530.5) mL/hour (2.5% (66.5%) of the total caspofungin clearance). The CLHD calculated from C_in_ − C_out_ amounted to 5.5 (1,550.5) mL/hour. The authors concluded that the influence of CRRT on caspofungin elimination appears to be negligible, and the standard dosage is probably appropriate for ICU patients on CRRT.

**Table 1:** Comparison of pharmacokinetic parameters of echinocandins in patients receiving and not receiving continuous renal replacement therapy

		**Pharmacokinetic parameter**				
		**C**_**max**_**, μg/mL**	**AUC**_**0-24h**_**, μg·hour/mL**	**t**_**1/2**_**, hours**	**CL**_**T**_	**V**_**d**_
PK studies in patients not receiving CRRT	Caspofungin [69]^a^	12.04	96.01	9.29	0.59 L/hour	N/A
	Anidulafungin [70]^b^	7.2	110.3	24-26	1.0 L/hour	34.5 L
	Micafungin [71]^c^	7.1	59.9	13.0	1.25 L/hour	23.0 L
PK studies in patients receiving CRRT	Weiler *et al*. [42]^d^					
	Single doses (CVVHF)	8.4	91	11.7	7.8 mL/hour per kg	138 mL/kg
	Single doses (CVVHD)	8.2	76	12.4	9.8 mL/hour per kg	175 mL/kg
	Single doses in patients not receiving CRRT	7.3	58	9.5	10.8 mL/hour per kg	135 mL/kg
	Steady-state (CVVHF)	11.0	107	12.4	5.3 mL/hour per kg	97 mL/kg
	Steady-state (CVVHD)	10.8	141	15.2	4.2 mL/hour per kg	89 mL/kg
	Steady-state in patients not receiving CRRT	8.8	100	12.6	4.9 mL/hour per kg	104 mL/kg
	Leitner *et al*. [36]^e^	8.5	109.9	28.8	NR	42 L
	De Rosa *et al*. [72]^f^	5.68-9.04	67.48-98.18	15.34-31.99	1.48-2.6 L/hour	32.81-48.48 L
	Kishino *et al*. [73]^g^	6.31	50.04	13.63	0.59 L/hour	11.53 L
	Hirata *et al*. [43]^h^					
	Patients receiving CRRT	N/A	N/A	N/A	1.4 L/hour	17.5 L
	Patients not receiving CRRT	N/A	N/A	N/A	1.4 (L/hour	16.2 L

Data are derived from different sources and trials and therefore are not always directly comparable. ^a^Pharmacokinetic data following single doses of 70 mg of caspofungin. ^b^Pharmacokinetic data following loading dose of 200 mg and maintenance dose of 100 mg of anidulafungin. ^c^Pharmacokinetic data following single doses of 100 mg of micafungin. ^d^Pharmacokinetics of caspofungin (single doses and steady-state) in critically ill patients on continuous venovenous hemofiltration (CVVHF), continuous venovenous hemodialysis (CVVHD), and control group not on continuous renal replacement therapy (CRRT). ^e^Pharmacokinetics of anidulafungin during CVVHF. ^f^Pharmacokinetics of anidulafungin in two critically ill patients with septic shock undergoing CVVHF. ^g^Pharmacokinetics of 50 mg of micafungin (steady-state) in living donor liver recipients receiving CVVHF. ^h^Pharmacokinetics of micafungin (150-300 mg) in patients on continuous venovenous hemodiafiltration and not receiving CRRT. AUC_0–24_, area under the curve over 24-hour dosing interval; CL_T_, total clearance (L/h or mL/hour per kg); C_max_, maximum concentration; N/A, not available; NR, not reported; PK, pharmacokinetic; t_1/2_, elimination half-life; V_d_, volume of distribution (L or mL/kg).

## Anidulafungin

In 2011, Leitner and colleagues [36] provided data on the adsorptive capacity of hemofilters for echinocandins from a study of 10 patients undergoing CVVHF for acute kidney injury by using a 1.2 m^2^ polysulfone hemofilter. The ultrafiltration rate was 25 mL/min. Anidulafungin was infused on 3 consecutive days, starting with a loading dose of 200 mg on day 1, followed by doses of 100 mg/day. On days 1, 2, and 3, blood samples were taken from the arterial and venous lines of the extracorporeal circuit before the start and at the end of the infusion as well as at 2, 4, 6, 8, and 24 hours after infusion; at the same time points, ultrafiltrate samples were collected. The concentration of anidulafungin in plasma and ultrafiltrate was assessed by high-performance liquid chromatography. Eight patients completed the scheduled 3-day CVVHF treatment. Pre-filter C_max_ (8.5 ± 3.6 mg/L) was reached 3 hours after the start of infusion. After the 100 mg dose on days 2 and 3, C_max_ values were 6.5 ± 3.1 mg/L and 5.9 ± 2.0 mg/L, respectively. Mean minimum concentration (C_min_) values were 3.1 ± 1.5, 3.0 ± 1.0, and 2.9 ± 1.1 mg/L at 24, 48, and 72 hours, respectively. Maximal differences in anidulafungin concentrations between the venous and arterial port (AV differences) were measured at 2 hours (19% ± 6%); AV differences steadily decreased to 14% ± 4% at 24 hours, 10% ± 2% at 48 hours, and 9% ± 2% at 72 hours. The mean arterial AUC_0-24_ was 109.9 ± 49.82 mg · h/L, the total clearance was 1.08 ± 0.41 L/hour, the volume of distribution was 41.97 ± 22.64 L, and the elimination half-life was 28.78 ± 10.40 hours.

Serum concentrations remained high at all times (C_min_ = 1.54 mg/L) and were similar to those in healthy adult subjects and in patients with fungal infections.

The authors concluded that pharmacokinetics of anidulafungin during CVVHF resembled findings in healthy adults and in adults with fungal infections and they recommend loading doses of 200 mg intravenous anidulafungin on the first day and 100 mg on consecutive treatment days in patients during CVVHF.

Similar results were obtained by De Rosa and colleagues [72]. In this study, the pharmacokinetics of standard doses of anidulafungin were studied in two ICU patients with candidemia and septic shock undergoing CVVHF. Both patients had satisfactory parameters of C_max_ (9.04 and 5.68 mg/L, respectively), area under the curve (AUC; 95.18 and 67.48 mg/L hour), and C_min_ (2.61 and 1.43 mg/L). AUC/MIC ratio and C_max_/MIC values were 11,887 and 8,435 (patient 1) and 1,130.25 and 710 (patient 2), respectively. According to these data, the authors concluded that anidulafungin presents only mild pharmacokinetic changes in septic shock patients undergoing CVVHF and no dose changes are needed.

## Micafungin

In 2004, Kishino and colleagues [73] reported the first data on the use of micafungin and CRRT. These authors studied six living donor liver recipients, four of whom underwent CVVHF. Micafungin (40 to 50 mg) was administered once daily for 3 weeks after liver transplantation. On the third day after the start of the infusion, several blood samples were taken from points located proximal and distal to the hemofilter. Ultradiafiltrate samples were also collected. CVVHF was performed by using a 1.5-m^2^ cellulose dialyzer. The ultrafiltrate was constantly obtained at 2,000 mL/hour by the post-dilutional method. The mean C_max_ and C_min_ (trough) values of micafungin in plasma were 6.31 ± 1.08 and 1.65 ± 0.54 μg/mL, respectively. The mean micafungin C_in_ − C_out_ were very similar. The mean micafungin C_in_ − C_out_ and the clearance of micafungin were 0.96 ± 0.04 and 0.054 ± 0.04 mL/min per kg, respectively, with 1.0 mg of the drug in the ultrafiltrate. The authors concluded that CVVHF had little effect on micafungin kinetics, and no dose adjustment or modification of dosing interval was needed during CVVHF.

Hirata and colleagues [43] evaluated the influence of continuous hemodiafiltration (CHDF) on the pharmacokinetics of micafungin in ICU patients. The authors studied four ICU patients receiving CHDF and nine ICU patients not receiving CHDF. CHDF was performed using a polymethyl methacrylate hollow-fiber membrane. Replacement fluid was delivered after the membrane into the venous limb of the circuit at a rate appropriate for the requirements of each patient. Standard dialysate was delivered at a rate of between 500 and 1,000 mL/hour. Blood samples were obtained before and after filtering, and urine samples and ultradiafiltrate samples were also collected for a steady-state assay of micafungin. The concentration of micafungin in serum was determined by high-performance liquid chromatography. Micafungin (dosage range, 150 to 300 mg/day) was administered to patients regardless of whether they were receiving CHDF. Finally, micafungin concentrations were available in three of the patients undergoing CHDF. In these three patients, mean micafungin concentrations at the inlet and outlet of the CHDF circuit were 12.7 ± 10.2 and 12.3 ± 10.1 mg/mL, respectively. Micafungin was not detected in the ultradiafiltrate, and urine concentration was 0.2 ± 0.1 mg/mL. The authors concluded that CHDF does not affect the pharmacokinetics of micafungin, so it is not necessary to adjust the micafungin dose in patients undergoing CHDF.

## Clinical significance of echinocandin drug adsorption

In an assessment of the clinical significance of drug adsorption, five points must be considered.

First, it is essential to know the pK/pD of the antibiotic [74,75]. For drugs that demonstrate time-dependent activity, the antimicrobial effect is related to the length of time that plasma concentrations are above a threshold MIC. For those that exhibit concentration-dependent activity, the antimicrobial effect is related to the post-distribution C_max_ or the area under the concentration-time curve. If the area under the inhibitory curve is the most important parameter and the adsorption is completely reversible, adsorption should not affect antimicrobial activity. In contrast, when the C_max_/MIC ratio is the best pK/pD parameter for this antibiotic, even if the adsorption is reversible, the C_max_ will be lower and antimicrobial activity could be compromised.

Second, to predict the clinical significance, it is important to know the time course of adsorption and whether this adsorption is reversible. The length of time that a filter has been used may affect its adsorption. We should determine the extent of adsorption at first dose of antibiotic given to the patient when starting CRRT with a new filter and the effect of repeated doses over an already-saturated filter when the steady-state drug concentration has been reached.

Third, the absorption of a drug could differ significantly depending on the filter membrane material and surface area. In general, adsorption onto PAN filters is higher than onto other synthetic membranes [59,60].

Fourth, the mode of CRRT could affect filter absorption capacity. A transmembrane pressure increase due to convection during hemofiltration would increase the hollow-fiber membrane’s adsorption capacity compared with the capacity observed in hemodialysis. This is especially relevant in high-flux convection [67,68].

Fifth, it is unclear whether differences in plasma pH and albumin concentration could affect drug adsorption. Antimicrobial activity depends on the fraction of unbound drug, and this fraction can be altered by many factors, such as systemic pH, heparin therapy, hyperbilirubinemia, concentration of free fatty acids, relative concentration of drug and protein, and the presence of uremic products and other drugs with competitive mechanisms [66].

In light of the points discussed above, some comments about these studies follow. From the study on caspofungin [42], as the authors noted, the different extracorporeal clearances obtained by estimation from C_in_ − C_out_ and from S_c_ suggest some adsorption of caspofungin by the hemofilter membrane. However, this adsorption appears to have no detectable effect on caspofungin plasma pharmacokinetics.

The study by Leitner and colleagues [36] demonstrates that anidulafungin adsorption reaches a plateau phase within 2 hours after first dosing. As a result, C_max_ is likely to be reduced. A mean total amount of 19% ± 6% of the anidulafungin in the arterial line was retained by absorption at the hollow-fiber dialyzer 2 hours after the first drug infusion. This adsorption to the polyethylene sulfone hemofilter seems to be saturable, and adsorption decreased over time. The authors concluded that there is no significant anidulafungin adsorption during CVVHF, so a standard dose can be recommended for patients on hemofiltration.

Some factors in the study by Kishino and colleagues [73] might influence the validity of their results with respect to the adsorption of micafungin. Micafungin concentrations were determined on the third day after the start of the infusion, when drug concentrations had already reached the steady state. Although the authors do not report whether the filter had been changed between the start of infusion and determinations, it is likely that it had not, so the membrane may very well have been completely saturated on the third day. Moreover, the cellulose membrane used has a low adsorptive capacity, and owing to poor biocompatibility in critically ill patients, cellulose membranes are no longer used in acute kidney injury. Finally, blood and ultrafiltration flows were low. All these factors could help explain why they found no drug loss due to adsorption.

The study by Hirata and colleagues [43] also has some methodological flaws. First, their data came from only three patients, and each of these apparently received different doses of micafungin. Moreover, replacement and dialysis flow varied based on the requirements of each patient. Finally and most importantly, the authors do not report how long the filters had been in use at the time of micafungin infusion or the number of doses administered before the concentration of the drug was determined.

## Final considerations

The differences reported by the studies in the amount of adsorption of echinocandins onto the hemofilter membrane could result in controversy about the clinical significance of echinocandin adsorption. However, the discrepant results are most likely due to the disparate conditions and methodologies of the studies rather than to the differences between echinocandin molecules. Given the great similarities in the structure and protein binding of the echinocandins, it is highly likely that these three molecules would interact identically with the different hemofilter membranes. If comparative studies among the three echinocandins were done under the same circumstances (same mode of renal replacement, same membrane and surface area, same flows, and same times of determination with respect to the infusion of the drug and the hemofilter life), the amount of drug lost by adsorption would very probably be nearly identical. This proportion of drug (possibly up to 20% in the first dose) does not seem to be clinically relevant, at least not for anidulafungin and caspofungin, because these two drugs are administered in a loading dose to reach steady-state concentrations on the first day. However, in micafungin, the proportion of drug lost may be clinically relevant because, unlike the other two echinocandins, micafungin is not administered in a loading dose [2]. Thus, the plasmatic levels on the first day might be compromised by drug loss due to adsorption in a hemofilter that is not yet saturated, especially when high-adsorptive capacity membranes and medium- to high-volume convection flows are used, as is recommended in critically unstable patients with severe sepsis and multiorgan dysfunction. Given the above, the removal of echinocandins by adsorption to the synthetic surfaces of hemofilters is unlikely to have clinical relevance once the steady-state concentration is reached.

For all echinocandins, it is unknown whether repeated hemofilter clotting and filter half-life of less than 24 hours could affect the fungicidal activity of echinocandins, so further studies are required. One major limitation in all of the studies referred to is the small size and the heterogeneity of the study population. Moreover, it is essential to use a validated technique to measure drug concentrations because the difference between pre- and post-filter concentrations may be very small, so caution is warranted in all studies.

## Conclusions

The removal of echinocandins by adsorption to the synthetic surfaces of hemofilters is unlikely to have clinical relevance once the steady-state concentration is reached.

## Abbreviations

AUC: area under the curve; AUC0-24: area under the curve over 24-hour dosing interval; AV: arteriovenous; CHDF: continuous hemodiafiltration; Cin − Cout: concentrations in the hemofilter/dialyzer inlet and outlet; CLHD: hemodialysis clearance; CLHF: hemofiltration clearance; Cmax: maximum concentration; Cmin: minimum concentration; CRRT: continuous renal replacement therapy; CVVHD: continuous venovenous hemodialysis; CVVHF: continuous venovenous hemofiltration; MIC: minimum inhibitory concentration; PAN: polyacrylonitrile; pD: pharmacodynamic; pK: pharmacokinetic; Sc: sieving coefficient; Sd: saturation coefficient.

## Competing interests

FJGdM has received honoraria from Astellas (Tokyo, Japan), MSD (Whitehouse Station, NJ, USA), Pfizer (New York, NY, USA), and Gambro-Hospal (Lund, Sweden). MAM-A declares that he has no competing interests. RF has received honoraria from MSD and Pfizer.

## Authors’ contributions

All authors made a substantial contribution to the manuscript. MAM-A and RF helped to review the literature and critically revised the manuscript for important intellectual content. FJGdM helped to review the literature and drafted the manuscript. All authors read and approved the final manuscript.
